# The consequences of stress on the brain and fear

**DOI:** 10.7554/eLife.108480

**Published:** 2025-08-06

**Authors:** Nicole C Ferrara, Sydney Trask

**Affiliations:** 1 Center for Neurobiology of Stress Resilience and Psychiatric Disorders, and the Department of Physiology and Biophysics, Chicago Medical School, Rosalind Franklin University of Medicine and Science North Chicago United States; 2 Department of Psychological and Brain Sciences, Indiana University Bloomington United States

**Keywords:** behavioral neuroscience, learned fear response, unlearned fear response, stress-enhanced fear learning, stress-induced sensitization of unlearned fear, paraventricular thalamus, Mouse

## Abstract

A region of the brain called the paraventricular thalamus mediates the impact of stress on unlearned fear responses, but is not involved in learned fear behavior.

**Related research article** Nishimura KJ, Paredes D, Nocera NA, Aggarwal D, Drew MR. 2025. Paraventricular thalamus hyperactivity mediates stress-induced sensitization of unlearned fear but not stress-enhanced fear learning (SEFL). eLife **14**:RP107670. doi: 10.7554/eLife.107670.

What is your biggest fear? Some of us fear heights, while others fear water or even birds, but we all respond with a range of behaviors when faced with something we fear (Fanselow et al., 2019). Moreover, stress can exaggerate fear behavior, and disentangling the impact of stress on fear behaviors has been a focus of research in behavioral neuroscience for a number of years (Long and Fanselow, 2012; Hoffman et al., 2014; Ponomarev et al., 2010).

Our fear behaviors are often learned, originating from an association between a neutral cue and a harmful stimulus, such that subsequent encounters with the previously neutral cue will elicit a fear response. In rodents, this can be a neutral environment paired with an electric shock to the foot, or an auditory stimulus paired with an electric shock. Learned fear behaviors are typically adaptive and critical for survival, allowing us to respond appropriately when cues predict that a harmful stimulus is likely. Traumatic or stressful events can also result in a heightening of learned fear responses, a process known as stress-enhanced fear learning (SEFL; Long and Fanselow, 2012). Furthermore, stress can give rise to unlearned fear behaviors in which, for example, an animal will freeze in response to an innocuous sound that it has not heard before (Hassien et al., 2020).

Changes in learned and unlearned fear responses are common in psychiatric conditions, including posttraumatic stress disorder (Norrholm et al., 2011; Parsons and Ressler, 2013). However, unlearned fear responses are especially difficult to target therapeutically because we do not know what causes such responses. Another challenge is to retain the adaptive aspects of fear while removing the negative impacts of stress.

Now, in eLife, Kenji Nishimura (University of Texas at Austin), Michael Drew (also at UT Austin) and colleagues – including Denisse Paredes (UCLA), Nathaniel Nocera and Dhruv Aggarwal (both at UT Austin) – report the results of a series of experiments that explore the effect of stress on both learned and unlearned fear behavior. They show that a region of the brain called the paraventricular thalamus mediates the impact of stress on unlearned fear (Nishimura et al., 2025).

In an initial experiment, Nishimura et al. placed mice in a novel environment and subjected them to four short electric footshocks; this group of mice was called the Stress group. Other mice were then placed in the same test environment but were not given footshocks; this was the No Stress group. The researchers then performed three behavioral tests. First, to explore the unlearned fear response, the mice were placed in a novel chamber, and exposed to three novel 30 second tones. Here, mice in the Stress group displayed higher levels of freezing to the tones than the mice in the No Stress group. Second, the researchers then measured the length of time the mice spent freezing when placed back in the test environment. As expected for learned fear, the mice in the Stress group, who received prior footshocks, displayed higher levels of freezing than the mice in the No Stress group. Third, to explore the impact of stress on learned fear, all the mice were placed in a novel test environment and given a single footshock that was of lower intensity than the original footshocks. Typically, a single low-intensity footshock like this would result in minimal freezing to that environment during later testing. However, when the mice were placed back in this environment the following day, those in the Stress group (the mice that had received four footshocks) exhibited higher levels of freezing than those in the No Stress group, indicating that prior stress enhanced fear learning. Interestingly, Nishimura et al. found no correlation between the learned and unlearned fear responses.

To explore further, the researchers used c-Fos immunohistochemistry to map cellular activity in six regions of the brain, and found that just one of these regions – the paraventricular thalamus – displayed increased activity following the tone test that was used to explore the unlearned fear response. Then, using an approach called fiber photometry, they demonstrated that neural activity in the paraventricular thalamus increased during the unlearned fear response, but not during the learned fear response.

Nishimura et al. then employed a neural manipulation technique called DREADDs (short for designer receptors exclusively activated by designer drugs) to manipulate the activity of the paraventricular thalamus as they repeated some of the experiments described above. When DREADDs were used to *reduce* the activity of the paraventricular thalamus during the tone test, there were also substantial decreases in the levels of fear displayed by the mice. In a complementary experiment, when DREADDs were used to *excite* the paraventricular thalamus in the absence of footshock stress, there was an increase in the levels of fear in response to the novel tone (i.e., the level of unlearned fear increased), but the level of learned fear did not increase. The paraventricular thalamus clearly has an important role in the unlearned fear response, but not in learned fear behavior (Figure 1).

**Figure 1.**
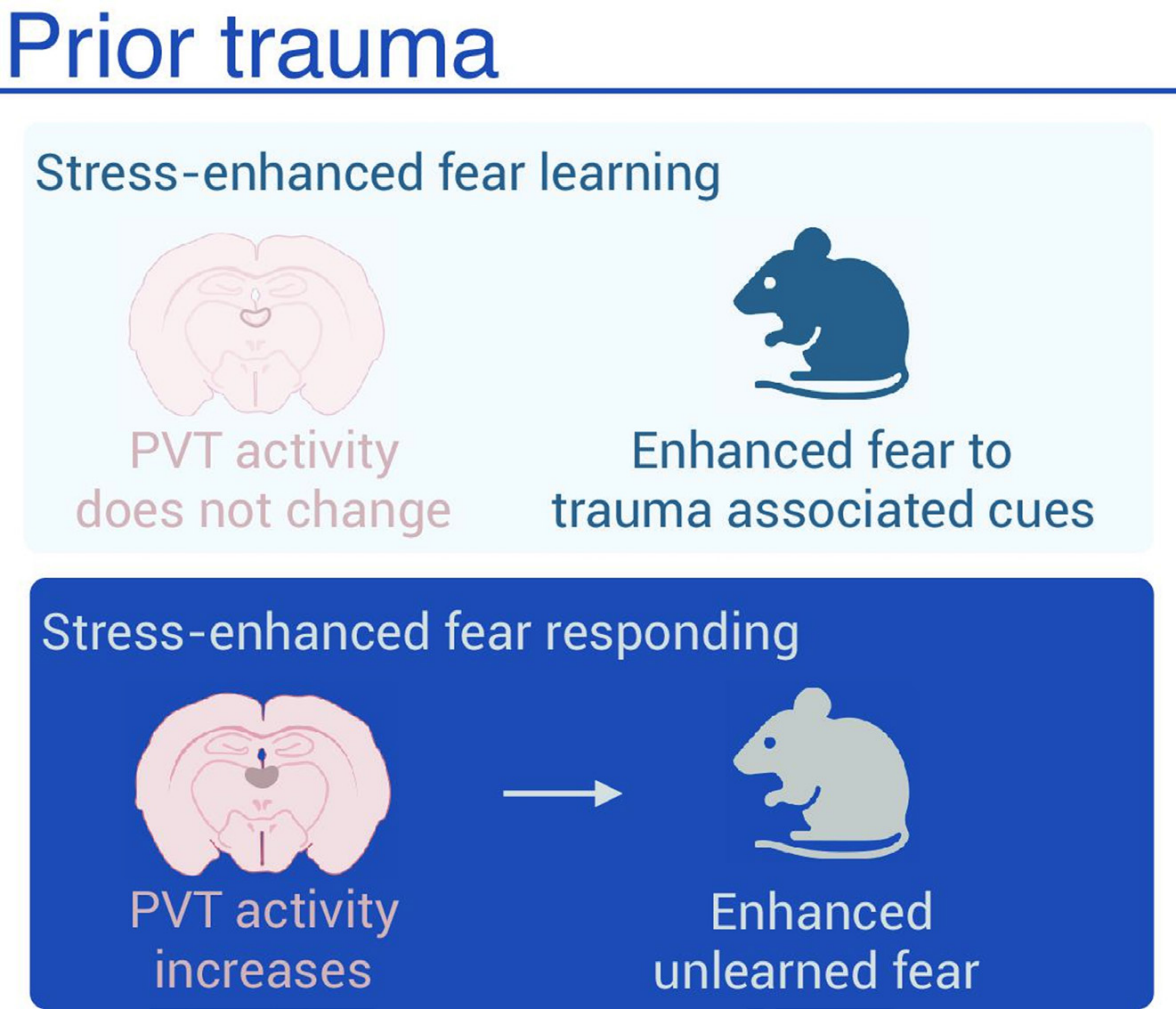
The impact of stress on fear. Learned fear is associated with cues that predict trauma, and the level of fear experienced can be increased by exposure to stress, a phenomenon known as stress-enhanced fear learning (top). Unlearned fear is not associated with trauma-predicting cues but, similar to learned fear, the level of fear experienced can be increased by exposure to stress, a phenomenon known as stress-enhanced fear responding (bottom). Nishimura et al. have shown that region of the brain called the paraventricular thalamus (outlined in red) is involved in unlearned fear but not learned fear. Created with BioRender.com.

This work of Nishimura et al. identifies behavioral and neural responses that regulate stress-potentiated unlearned fear, although our understanding of the circuit, cellular, and molecular mechanisms involved remains much less advanced than our understanding of the mechanisms responsible for the impact of stress on learned fear behavior. Future work should also explore the behavioral conditions that produce stress-enhanced unlearned fear, as well as determine if this effect is specific to auditory stimuli, and seek to understand why sex seems to impact fear responses in humans despite being absent in the present experiments (Inslicht et al., 2013; Ressler et al., 2011).
